# Environmental Particulate (PM2.5) Augments Stiffness-Induced Alveolar Epithelial Cell Mechanoactivation of Transforming Growth Factor Beta

**DOI:** 10.1371/journal.pone.0106821

**Published:** 2014-09-16

**Authors:** Marilyn M. Dysart, Boris R. Galvis, Armistead G. Russell, Thomas H. Barker

**Affiliations:** 1 The Wallace H. Coulter Department of Biomedical Engineering, Georgia Institute of Technology and Emory University, Atlanta, Georgia, United States of America; 2 The School of Civil and Environmental Engineering, Georgia Institute of Technology, Atlanta, Georgia, United States of America; 3 The Parker H. Petit Institute for Bioengineering and Biosciences, Georgia Institute of Technology, Atlanta, Georgia, United States of America; University of California, San Diego, United States of America

## Abstract

Dysfunctional pulmonary homeostasis and repair, including diseases such as pulmonary fibrosis (PF), chronic obstructive pulmonary disease (COPD), and tumorigenesis have been increasing over the past decade, a fact that heavily implicates environmental influences. Several investigations have suggested that in response to increased transforming growth factor - beta (TGFβ) signaling, the alveolar type II (ATII) epithelial cell undergoes phenotypic changes that may contribute to the complex pathobiology of PF. We have previously demonstrated that increased tissue stiffness associated with PF is a potent extracellular matrix (ECM) signal for epithelial cell activation of TGFβ. The work reported here explores the relationship between tissue stiffness and exposure to environmental stimuli in the activation of TGFβ. We hypothesized that exposure of ATII cells to fine particulate matter (PM2.5) will result in enhanced cell contractility, TGFβ activation, and subsequent changes to ATII cell phenotype. ATII cells were cultured on increasingly stiff substrates with or without addition of PM2.5. Exposure to PM2.5 resulted in increased activation of TGFβ, increased cell contractility, and elongation of ATII cells. Most notably, on 8 kPa substrates, a stiffness greater than normal but less than established fibrotic lung, addition of PM2.5 resulted in increased cortical cell stiffness, enhanced actin staining and cell elongation; a result not seen in the absence of PM2.5. Our work suggests that PM2.5 exposure additionally enhances the existing interaction between ECM stiffness and TGFβ that has been previously reported. Furthermore, we show that this additional enhancement is likely a consequence of intracellular reactive oxygen species (ROS) leading to increased TGFβ signaling events. These results highlight the importance of both the micromechanical and biochemical environment in lung disease initiation and suggest that individuals in early stages of lung remodeling during fibrosis may be more susceptible than healthy individuals when exposed to environmental injury adjuvants.

## Introduction

Dysfunctional pulmonary homeostasis and repair, including diseases such as pulmonary fibrosis (PF), chronic obstructive pulmonary disease (COPD), and tumorigenesis, have been steadily increasing over the past decade. Many fibrotic pathologies are characterized by excessive extracellular matrix (ECM) deposition, interstitial scar tissue formation, and an increase in tissue stiffness. Specifically, during the course of pulmonary fibrosis, functional lung tissue of the alveoli is replaced with collagen-rich ECM, which leads to rapid and severe decreases in lung compliance and irreversible loss of lung function [1], [2]. In addition, another hallmark of PF and other fibrotic conditions is the influx of contractile myofibroblasts. This influx of myofibroblasts further perpetuates the disease through persistent matrix production and contraction. Myofibroblasts are recruited from a variety of sources including local mesenchymal cells, bone marrow progenitors, and via a process of epithelial to mesenchymal transition (EMT), where epithelial cells transdifferentiate into fibroblast like cells. Once these fibroblasts become activated, they transform into myofibroblasts that are capable of secreting ECM components. During wound healing, myofibroblasts lay down a temporary matrix that epithelial cells migrate over to repair the damaged tissue. Fibrosis is thought to occur when this process becomes dysregulated, resulting in persistent matrix production and the formation of a scar.

Lack of effective treatment options for this disease, and other fibrotic pathologies is largely due to the lack of understanding of the exact mechanisms that initiate and propagate fibrosis, however mounting evidence suggests that apoptotic signaling of the lung “precursor” cell, the type II alveolar epithelial cell (ATII), contributes to the initiation and progression of these diseases [3]–[7]. ATII cells are pseudo cuboidal, multifunctional cells that are considered the “protector of the alveolus” due to their central role in defense and repair. ATII cells act as the primary surfactant secreting cells, precursors to ATI cells, and in many cases as non-professional antigen presenting cells [8]. These various functions underscore the importance of these cells in maintaining pulmonary function. During normal repair, ATII cells are thought to proliferate, migrate onto a provisional matrix and differentiate into ATI cells. However, recent evidence has suggested that repeated injury of ATII cells may be an underlying contributor of pulmonary fibrotic disorders [8], [9]. Specifically, ATII cells can change their phenotype in response to several stimuli including active transforming growth factor beta (TGFβ), tumor necrosis factor alpha (TNFα), epidermal growth factor (EGF), and reactive oxygen species (ROS), as well as in response to ECM proteins including fibronectin (Fn) [5], [10]–[17]. The role of TGFβ is most well defined of all these factors and has been highly implicated in the onset and progression of fibrosis by inducing ATII cell EMT as well as through activation of resident fibroblasts. TGFβ induced EMT may directly contribute to the disease by increasing the number of mesenchymal, ECM secreting cells. Additionally, ATII TGFβ signaling to neighboring fibroblasts may result in their activation, again increasing the number of ECM secreting myofibroblasts. Although the exact mechanisms of action are still unknown, it is under agreement that TGFβ is a major player, and thus understanding what factors lead to increased TGFβ activation and signaling may provide new insights to the development of treatments for PF.

TGFβ must be activated in order to bind to its receptor(s), which can be performed through several different mechanisms including the presence of ROS, proteolytic cleavage, decreased pH, and physical forces [17]–[34]. The ability of ROS to activate TGFβ is of particular interest in the lung due to the continued exposure of the lung to insults of injury, including environmental particulate matter (PM). Environmental PM is capable of depositing in the airways, penetrating into the alveoli, increasing respiratory distress, and then exacerbating pre-existing pulmonary conditions. Previous studies have highlighted the potential role of PM exposure in predisposing patients to asthma, bronchitis, COPD, and resulting fibrosis [35]–[38]. The fibrogenic potential of PM is likely due to several factors including particle size, surface area, and composition. Smaller particles in the 1 to 10 micron range have been shown to reach the distal lung, resulting in a higher potential to cause injury due to their high surface to mass ratio [38]–[40]. Furthermore, particle composition is likely an important determinant of the effect of the PM on the progression of pulmonary disease. Both organic and inorganic agents, such as transition metals, hydrocarbons, and endotoxins can contribute to the composition of the PM [40]–[42]. Previous studies have shown that many of these components of PM can induce cellular damage, which can stimulate fibrotic remodeling pathways [43]–[46].

Furthermore, recent evidence indicates that cells sense and respond to the composition and mechanical properties of their underlying ECM. This is of particular interest when studying diseased states, such as PF, where significant changes to both the biochemical and biophysical properties of the ECM have been observed. Specifically, in the case of PF, the ECM is known to shift biochemically from a predominantly laminin (Ln) and elastin matrix to a provisional matrix that is predominantly composed of Fn and eventually collagen. These differences in underlying matrix composition have been shown to have a dramatic effect on the phenotype of alveolar epithelial cells, where Ln maintains an epithelial phenotype and Fn drives EMT [5], [47]–[51]. Additionally, cells are extremely sensitive to their micromechanical environment, particularly the rigidity or stiffness of the matrix. Previous work has shown that matrix rigidity plays a critical role in directing many aspects of cellular behavior including stem cell, myoblast, and breast epithelial cell differentiation; cellular motility; contraction and focal adhesion formation, and it has also been shown to contribute or prevent a malignant phenotype depending on the context [11], [16], [52]–[63]. The mechanical properties of the ECM can exert both biophysical and biochemical effects on resident cells. First, cells will actively engage their contractile machinery in order to “match” their internal stiffness with their extracellular environment [29], [54], [55], [64], [65]. Contraction of actin and myosin leads to force exertion on ECM proteins including latent TGFβ leading to its subsequent activation [26], [27], [66], [67]. Second, many ECM proteins, like Fn, have been theorized to display force mediated unfolding resulting in the exposure of cryptic, or hidden, sequences that then may influence cell phenotype. While the precise mechanism of cell rigidity sensing is still unclear, it likely involves the transduction of force-mediated signaling via cell contractility and activation of TGFβ in an intricate positive feedback loop. These facts imply a role for lung stiffness; associated with PF, in regulating cell behavior, possibly including PM-induced changes to ATII cell phenotype. To date, the interplay between the presence of lung disease, such as pulmonary fibrosis, and exposure to PM has yet to be studied. Here, we explore the contribution of ECM mechanics on pulmonary homeostasis and pathological progression by examining the relationship between increases in lung ECM stiffness, exposure to PM, and ATII cell phenotype.

## Materials and Methods

### Cell Isolation and maintenance

RLE-6TN cells, an alveolar type-II epithelial (ATII) cell line, were purchased from ATCC and maintained in DMEM/F12 media with 10% fetal bovine serum (FBS) and 1% penicillin/streptomycin (P/S) and incubated at 37°C at 5% CO_2_. Media was refreshed every 48 hours and cells were split upon reaching 95% confluency.

### Poly-acrylamide gel production

Poly-acrylamide (PA) gels of varying bisacrylamide concentrations were created on amino-silanated coverslips as previously described [68]. PA gel solutions were produced by combining acrylamide and bisacrylamide to final concentrations of 8% acrylamide (Biorad, Hercules, CA, USA) and 0.048%, 0.117%, 0.208%, or 0.260% bisacrylamide (Biorad) to obtain gels with final elastic moduli of 2 kPa, 8 kPa, 16 kPa, or 24 kPa, respectively. Fifty (50) µl of each solution was polymerized by the addition of ammonium persulfate (VWR, West Chester, PA, USA) and N,N,N′,N′-tetramethylethylenediamine (Biorad, Hercules, CA, USA) (1% and 0.1% final concentration respectively). The gels were allowed to polymerize for approximately 30 minutes, then washed three times with PBS. Fn was covalently attached to the surface using the heterobifunctional crosslinker sulfosuccinimidyl-6- (4′-azido-2′ nitrophenyl-amino) hexanoate (sulfo-SANPAH; Pierce Chemical Co., Rockford, IL., USA). Following an overnight incubation with the Fn, gels were washed three times with PBS.

### Fine particulate matter isolation

Fine particulate matter samples were collected on Teflon filters from the Atlanta area (Dekalb county) as part of a study for the Georgia Tech Civil and Environmental Engineering department. The samples were collected daily using multichannel particle composition monitors (PCM). The specific area of collected PM is located near two major highways (I20 and I285), and near a school, both of which can lead to increased levels of mobile source generated particulate matter [8]. In each channel of the PCM, air first passes through a cyclone separator to remove particles that are greater than 10 um. In one channel, the air next passes through a WINS impactor to remove particles that are greater than 2.5 µm. Finally, the remaining particles (i.e. the PM2.5 fraction) is collected on a Teflon filter. In channel two, after passing though the cyclone, the air passes through an annular denuder to remove acidic and alkaline gases. Then the air passes through the WINS impactor and the remaining particles are captured on a nylon filter in order to analyze the ionic species. In the third channel, the air passes through a denuder in order to remove organic gases, flowed by the WINS impactor, and the particles are collected in a quartz filter for organic and elemental carbon analysis [8], [9]. After collection in the particle composition monitors, the filters are stored at −20°C for later chemical analysis. The Teflon filter is stored for exploratory analyses, such as this study. In order to prepare the PM2.5 fraction for cell culture experiments, five different PM containing Teflon filters were weighed to determine the total particulate mass on each filter. Five samples were pooled together to minimize variation based on the day collected, and placed in sterile 50 ml centrifuge tubes fully covered with 5 ml of diH_2_0, and sonicated 5 times for 10 minutes to release the particles captured on the filters. The samples were filtered through a 5 µm diameter pore size filter to remove any larger particle debris. The pooled sample was resuspended in DMEM/F12 culture media to a final concentration of 100 µg/ml.

### LIVE/DEAD assay of cell viability

RLE-6TN cells were cultured for 24 hours with increasing concentrations of PM2.5 ranging from .01 µg/cm^2^ to 50 µg/cm^2^ and cell viability was determined by staining with trypan blue. Cells were trypsinized and then resuspended in a 1∶1 suspension using 0.4% trypan blue. The cell suspension was analyzed by counting dead cells (stained blue) vs. total cells using a hemacytometer. Each concentration of PM2.5 was tested in triplicate and results are presented as% viable cells for each PM concentration.

### Cell culture experiments

Serial dilutions were performed from the 100 µg/ml stock to prepare experimental groups of 1∶100, 1∶1000, and 1∶10000 which correspond to physiologically relevant concentrations of approximately 10 µg/cm^2^, 1 µg/cm^2^, and 0.1 µg/cm^2^ respectively. RLE-6TN cells were maintained and passaged in DMEM/F12 media supplemented with 10% FBS+1% P/S. Cells were plated at a density of 100,000 cells/cm^2^ in growth medium in the absence or presence of PM. The particulate containing media was made fresh for each experiment, keeping total PM mass from the pooled filters consistent for each preparation. The fresh particulate containing media was then added directly to cell culture wells of varying substrate stiffnesses for subsequent different studies.

### TGFβ activation assay

RLE-6TN cells were cultured on Fn coated PA gels, Fn-coated glass, or Ln-coated glass as previously described [27], [29] with addition of particulate matter. TGFβ activation was determined by a mink lung epithelial cell (MLEC) assay as previously described [69]. MLECs stably transfected with an expression construct containing a truncated Pai-1 promoter fused to the firefly luciferase reporter gene respond in a dose dependent manner to active TGFβ, but are incapable of activating TGFβ. After 5 days of RLE-6TN culture on the various substrates, MLECs were added at a density of 50,000 cells/cm^2^ on top of the RLE-6TN cells in serum free DMEM/F12 media +1% BSA. Cells were co-cultured for 16 hours, lysed and luciferase activity was determined via the One-Glo luciferase assay (Promega). To determine total levels of TGFβ, samples were heated to 85°C for 10 minutes prior to plating of MLEC cells. To ensure PM alone did not activate TGFβ, soluble TGFβ was added along with PM as a control. In order to determine if substrate stiffness alone affected the sensitivity of the MLEC cells to active TGFβ, MLEC were cultured for 16 hours with the addition of 200 ρg/mL active TGFβ and analyzed for changes in luminescence. To determine the role of contractility, RLE-6TN cells were cultured with or without the addition of PM in the presence of 10 µM Y-27632 (EMD Biosciences). Additionally, to determine the effect of ROS on TGFβ activation, cells were cultured in the presence of 5 µM NAC. Luminescence was measured with a Synergy H4 Multi-Mode Plate Reader (BioTek, Winooski, VT, USA). Luminescence values were normalized to MLECs cultured in the absence of TGFβ. Levels of active or total TGFβ activation were then calculated through interpolation using a standard curve. Results presented are from 3 independent triplicate experiments.

### Immunofluorescence staining and cell shape analysis

Following culture for 5 days, cells were washed with PBS, fixed with 4% formaldehyde, permeabilized with 0.2% Triton-X 100 and then blocked with 10% goat serum. To characterize cell shape, actin was stained with Texas-red phalloidin (Invitrogen) and nuclei were stained with Hoescht stain (Invitrogen). Images were acquired with a Nikon Eclipse (TiE) inverted fluorescence microscope at 20× magnification (PlanFluor 20X, 0.5 NA objective) with a CoolSNAP HQ2 Monochromatic CCD camera. Experiments were performed in triplicate, and images presented are representative from 5–10 random fields for each independent experiment. Images were quantified by determining the total intensity of phalloidin staining in Image J and then normalizing to the number of nuclei per image. To characterize circularity, area and perimeter of individual cells stained for actin were determined for each condition using Image J (NIH Freeware) image processing software, then circularity was determined using the equation circularity  = 4π(area/perimeter^2^). Three independent images were analyzed for each condition, and at least 10 cells were analyzed per image. Data is pooled from all 3 images analyzed per condition.

### Atomic force microscopy (AFM) nanoindentation analysis

RLE-6TN cells with or without the addition of each of the three concentrations of PM2.5 were cultured on 8 kPa gels in a 0.17 mm thick glass-bottom petri dish (World Precision Instruments, Inc.). Using the Asylum MFP-3D-BIO AFM, single force points were measured from at least 5 peri-nuclear regions that were greater than 300 nm in height by force spectroscopy using contact mode at a scan rate of 1.2 Hz. The silicon nitride AFM tip (Veeco) was customized with a 4.74 µm diameter polystyrene bead to allow for imaging at appropriate resolution scales and for elastic modulus determination using the Hertz-contact model. Single cantilever's unique spring constants were determined using the thermal resonance frequency method, with values typically ranging 0.1–0.3 N/m. To determine the Young's modulus of each contact point, the Hertz-contact model for determining the elastic forces between two spheres was used (Eq. 1). Using known values from the AFM measurement (xs is scanner displacement; xc is the cantilever displacement; c.p. is the contact point) and predetermined values for the cantilever spring constant (k) and beaded-tip radius, the elastic modulus can be solved for (E) (bead force, FB, is equal to k* xc). The assumption of an isotropic material or that Poisson's ration (ν) is equal to 0.5 is valid for the small indentation depths under operation.
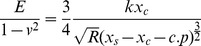
(1)


### DCFH_2_-DA oxidation assay for measuring ROS

RLE-6TN cells were cultured for 5 days with PM containing media with or without the addition of 5 µM NAC. Cells were washed with HBSS, and incubated with 10 µM DCFH_2_-DA for 30 minutes at 37°C to measure oxidation. Controls were included that were not incubated with any dye. To determine the role of TGFβ on intracellular ROS levels, cells were cultured in the presence of 10 µg/ml TGFβ neutralizing antibody (R&D Systems). Media with antibody was changed every 48 hours. After incubation, cells were washed in sterile HBSS and CM-DCFH_2_ fluorescence was measured (excitation of 485/20 nm filter and emission of 528/20 nm filter). Cells were then washed again and incubated with 5 µg/ml Hoescht 33342 for 30 minutes at 37°C. The Hoescht fluorescence was measured (excitation of 350 nm and an emission of 461 nm). The CM-DCFH_2_ fluorescence was normalized to the Hoescht reading to account for cell number.

### Statistical analysis

All statistical analysis for three or more experimental groups was performed by multivariate ANOVA, using Prism (GraphPad Software Inc., La Jolla, CA, USA). Statistical significance between groups was determined by performing Tukey's *post hoc* analysis. All data is presented as mean +/− SEM and statistical significance is achieved for p<0.05.

## Results

Exposure to fine particulate matter (PM2.5) (particulate matter with an aerodynamic diameter less than 2.5 um) has been associated with a number of pulmonary diseases including asthma, bronchitis, COPD and fibrosis. Previous studies have shown that small-inhaled particles, in the micron range, result in more toxic and fibrotic effects on alveolar cells due to their higher surface to mass ratio, which prevents the particles from being cleared from the lungs. In addition, different components of the particles are able to activate cytokines and growth factors, including TGFβ, a known regulator of ATII cell phenotype. To date, no studies have explored the effect of environmental PM in conjunction with substrate stiffness on ATII cell phenotype. We have previously shown that substrate stiffness induces changes to alveolar epithelial cell phenotype and may be a regulator of processes that drive PF [16], [70].

### Particulate Matter Isolation and Analysis

PM used for this study was collected from 1-March-2004 to 30-June-2004 using a filter based particle composition monitor (PCM) as part of the longer term Assessment of the Spatial Composition in Atlanta (ASACA) program [71]. The PCM is a 3 - channel system that collects 24 hour integrated samples for analysis of ionic, carbonaceous, and metallic species in the PM2.5 size range. The monitor is controlled by a data acquisition system (DAS) that activates sampling, sequences the filters and controls sample flow to a flow rate of 16.7 L/min through each channel. Filters were installed approximately one day prior to sampling and removed one day following exposure to ambient air. Ambient air was drawn through each channel of the monitor and then flowed through denuders for selective removal of gases, a WINS impactor for providing a 2.5 µm size cut, and finally the sampling media. The PCM was mounted approximately 2.5 m above ground [71]. In channel 1, the sampled air was passed through onto a single Teflon filter. PM2.5 captured on the Teflon filter is archived for exploratory analyses, such as this one. Ionic species including sulfate, nitrate, and ammonium were collected on a nylon filter using channel 2. Finally, channel 3 was used for quantification of elemental and organic carbon. Regular maintenance of the PCM was performed over the collection period.

### Particulate matter sample analysis

Concentrations of water-soluble sulfate, nitrate and ammonium, as well as elemental and organic carbon were determined from filter samples collected using the ASACA PCM. In addition, data on these species, as well as from elemental analyses, were available from the nearby (approximately 30 m) monitoring site run by the Georgia Environmental Protection Division (GaEPD), which is also reported as part of the US EPA Air Quality System (AQS). While the ASACA data (and the filters used) are available every day during the period, the GaEPD samping is done every third day. **Table 1** summarizes the average composition of these components from our filters as well as the data reported in AQS. Full data is shown in Supplemental Material. The ionic and carbonaceous composition of the PM obtained from the nylon and Teflon filters shows that the bulk of the PM was sulfate and organic carbon **(** **Table 1** **)**, with a significant fraction being elemental carbon. A more complete presentation of the PM composition, including elemental concentrations observed at the GaEPD site and a comparison of the average ionic and carbonaceous PM concentrations for daily (e.g., corresponding to the samples used in the cellular exposure study) versus 1-in-3 day sampling (as used for the source apportionment) is contained in Supplemental Material (Table S1).

**Table 1:** Average Comparison of AQS and ASACA Particle Composition for Species of Interest.

	SD AQS Data (ug/m3)					ASACA Data (ug/m3)				
	NH4	NO3	SO4	OC	EC	NH4	NO3	SO4	OC	EC
Average	1.22	0.73	3.86	4.70	0.95	1.40	0.65	4.12	4.92	0.80

### Source apportionment modeling of PM2.5

Measurements were used to conduct Chemical Mass Balance (CMB) source apportionment for the days that coincided with the samples used in this analysis as previously described [72] and where GaEPD data was available (i.e., every third day). The reason for conducting source apportionment when the GaEPD data is available is the need to have elemental species concentrations. The good agreement between the ASACA and GaEPD sampling, and their close proximity, suggest they are sampling from very similar air masses, and that the source apportionment conducted using the concentrations observed at the GaEPD site are applicable for the filters collected as part of the ASACA monitoring. On average, the most prominent sources for the resulting particulate matter were ammonium sulfate and bisulfate, largely due to coal burning in the region, which together comprised 38%, biomass burning at 20%, gasoline-fueled vehicles at 8%, and diesel vehicles at 9%. These results are summarized in **Table 2**, with full data in Supplemental Material. This source distribution is expected as coal is a major fuel-stock for electricity generation, the sampling site is near two freeways and the March-April months are when prescribed fires are most common in the Southeast.

**Table 2:** Average Source Apportionment for South Dekalb PM2.5 Collection.

	GV	DV	DUST	BURN	COAL	AMSULF	AMBSULF	AMNITR	SOC
AVERAGE	10.01%	6.86%	4.70%	11.06%	0.83%	18.65%	26.64%	9.06%	12.19%

Results from the particle analysis show that the composition was primarily sulfate, related ammonium, and organic carbon, with smaller amount of elemental carbon, and that the primary sources were those leading to sulfate, biomass burning and mobile sources.

### RLE-6TN cells are viable up to a concentration of 10 µg/cm^2^ of PM2.5

RLE-6TN cells were plates in 6 well plated at a density of 100,000 cells/cm^2^ and allowed to attach for 24 hours, then treated with increasing concentrations of isolated PM2.5 for 24 hours. PM2.5 was isolated from the Teflon filters and diluted in cell culture media to concentrations of 0.01 µg/cm^2^, 0.1 µg/cm^2^, 1 µg/cm^2^, 5 µg/cm^2^, 10 µg/cm^2^, 25 µg/cm^2^, and 50 µg/cm^2^. These concentrations were picked based on previous literature suggesting these concentrations are generally within physiologically relevant ranges and that anything over 100 µg/cm^2^ has been shown to be toxic to cells. After culture for 24 hours, the cells from each PM2.5 concentration group were trypsinized and resuspended in a 1∶1 suspension with trypan blue. The suspension was analyzed by counting dead cells, which were stained blue, and normalizing to the total number of cells in each group.

**Figure 1:**
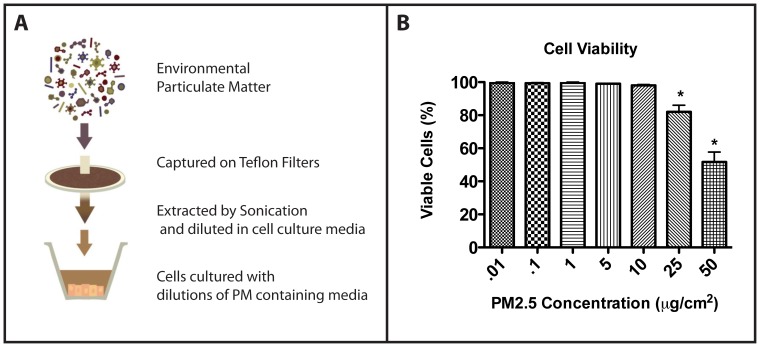
RLE-6TN cells are viable up to a concentration of 10µg/cm^2^. (A) Schematic illustrating process of PM2.5 collection for cell culture experiments. (B) RLE-6TN cells were cultured with increasing concentrations of isolated PM2.5 for 24 hours and analyzed using the trypan blue assay. Approximately 100% of cells were viable through a concentration of 10µg/cm^2^. Treatment with concentrations above 10µg/cm^2^ resulted in significant cell death. Significance shown for * (p<0.01)

As shown in **Figure 1**, over 99% of cells were viable up to a concentration of 10 µg/cm^2^. When the concentration was increased to 25 µg/cm^2^ and 50 µg/cm^2^, cell viability began to decline rapidly to only 83% and 56% of viable cells, respectively. From these viability results, a low, medium, and high concentration of PM2.5 exposure were chosen to explore in further studies. PM2.5 was isolated from the Teflon filters and added to cell culture media at 0.1 µg/cm^2^, 1 µg/cm^2^, and 10 µg/cm^2^ for each of the following described experiments. These concentrations closely mimic environmental exposures in rural (farm), urban city (Atlanta), and metropolis (Beijing) as reported by the World Health Organization.

### Addition of PM2.5 results in elongated cells and decreased circularity

**Figure 2:**
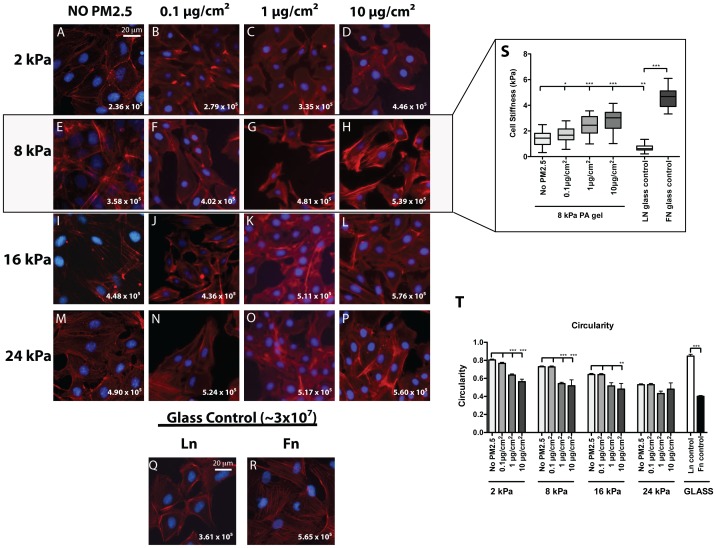
Exposure to PM2.5 results in elongated cell phenotype and increased cell cortical stiffness. RLE-6TN cells were cultured on Fn-PA gels or Fn- or Ln-coated glass for 5 days with the addition of 10µg/cm^2^, 1µg/cm^2^, or 0.1µg/cm^2^ concentrations of PM2.5, and changes in the actin cytoskeleton were analyzed by phalloidin staining of actin filaments and quantified as mean fluorescent staining of actin per cell (A-R). Single cell cortical stiffness of RLE-6TN cells cultured on 8 kPa gels were measured by AFM and significance shown for each group compared to the No PM2.5 control (S). Cell circularity was calculated from acquired images. Values closer to 1 indicate a more rounded, epithelial like cell (T). Experiments were performed in triplicate, representative images are presented and significance shown for * (p<0.05), ** (p<0.01) and *** (p<0.001).

In order to explore how exposure of RLE-6TN cells to PM2.5 in a range of different elastic moduli environments affects cell morphology, cells were cultured on PA gels of varying substrate stiffness (2–24 kPa) with or without the addition of PM2.5 at 0.1 µg/cm^2^, 1 µg/cm^2^, or 10 µg/cm^2^ for five days. This range of substrate stiffness was chosen based on previous literature that shows that ATII cells experience significantly greater stiffness in fibrotic versus normal lung, with an average Young's modulus of normal lung at approximately 2 kPa versus fibrotic lung at approximately 18 kPa [27], [73]. Additionally, during remodeling observed in PF, the underlying matrix shifts from a predominantly Ln and elastin matrix to one that is composed predominantly of Fn. Therefore to most closely mimic this environment, each of our PA gels was coated with Fn. Furthermore, Ln-coated glass, known to maintain epithelial phenotype, and Fn-coated glass, known to induce EMT, were used as controls as previously described [27], [29], [51]. The cells were then fixed and stained with Texas Red-X conjugated phalloidin to visualize any changes in the actin cytoskeletal organization. Cells cultured on soft substrates and on Ln-coated glass without the addition of PM2.5 displayed the typical rounded epithelial morphology and diffuse cortical staining for actin **(** **Figure 2A, Q** **).**

As substrate stiffness was increased, cells displayed a subsequent elongated morphology and thick, aligned actin filaments **(** **Figure 2I, M** **).** In addition, when PM2.5 was added to the cultures, most significantly at the 10 µg/cm^2^ concentration, cells exhibited enhanced staining of actin filaments. Notably, cells cultured on 8 kPa gels showed a considerable increase in actin fibers and cell elongation with the addition of PM2.5 when compared with matched substrate-stiffness controls **(** **Figure 2** **, E-H).** This substrate stiffness is of particular interest because it most closely matches that of slightly fibrotic lung tissue, indicating that PM2.5 may have more marked effects on a lung that is undergoing fibrotic remodeling. In addition, this increase appeared to be concentration dependent, with slightly more cell elongation and stress fiber formation seen at the 10 µg/cm^2^ concentration compared to 1 µg/cm^2^ and 0.1 µg/cm^2^ concentrations. Total fluorescent intensity of each image was calculated, normalized to the total number of nuclei, and reported in the bottom right corner of each image **(** **Figure 2** **A-R).** Additionally, cell circularity was calculated to quantify differences in the observed cell shape. Values closer to 1 indicate a more rounded, epithelial like cell ***(*** **Figure 2T** ***)***. Statistical significance is shown for each experimental group in comparison to its same substrate stiffness group with no PM2.5 added. These results suggest that the addition of PM2.5, most notably at the 10 µg/cm^2^ concentration result in a more elongated cell indicated by the decreased values of cell circularity.

### Increased cell elongation and stress fibers coincide with increased cell stiffness

Based on the observation that PM2.5 resulted in decreased cell circularity and increased actin fiber staining, most notably at the 8 kPa substrate stiffness, it was investigated if these phenotypic observations coincided with an increase in cell stiffness. Previous studies have linked increased epithelial cell stiffness in increased substrate stiffnesses environments that are commonly seen during PF [27]. Furthermore, several studies have linked increased cell stiffness with increased cell contractility and TGF β activation [27].

Therefore, to explore if the addition of PM2.5 increased cell stiffness, RLE-6TN cells were cultured on Fn-cross-linked 8 kPa gels with the addition of 10 µg/cm^2^, 1 µg/cm^2^, 0.1 µg cm^2^, or no PM concentrations of PM2.5 and cell stiffness was measured by atomic force microscopy (AFM). It was observed that there was a concentration dependent increase in cell-stiffness with the addition of increasing amounts of PM2.5 when compared to the 8-kPa control. When cells were cultured with the addition of either 10 µg/cm^2^ or 1 µg/cm^2^ concentrations of PM2.5, cortical cell stiffness was increased (average Young's Moduli of 2.8 kPa and 2.4 kPa, respectively) over cells that were cultured with the 0.1 µg/cm^2^ concentration of PM2.5 or no addition of PM2.5 (Average Young's moduli of 1.5 kPa and 1.4 kPa, respectively). Statistical significance is shown in comparison to the no PM2.5 added control **(** **Figure 2S** **)**. These results indicate that the addition of PM2.5 increases cell stiffness and agrees with the increase in cortical actin staining and decreased circularity observed **(** **Figure 2E-H, S,T** **)**.

### Alveolar epithelial cells display increased TGFβ activation with the addition of PM2.5

**Figure 3:**
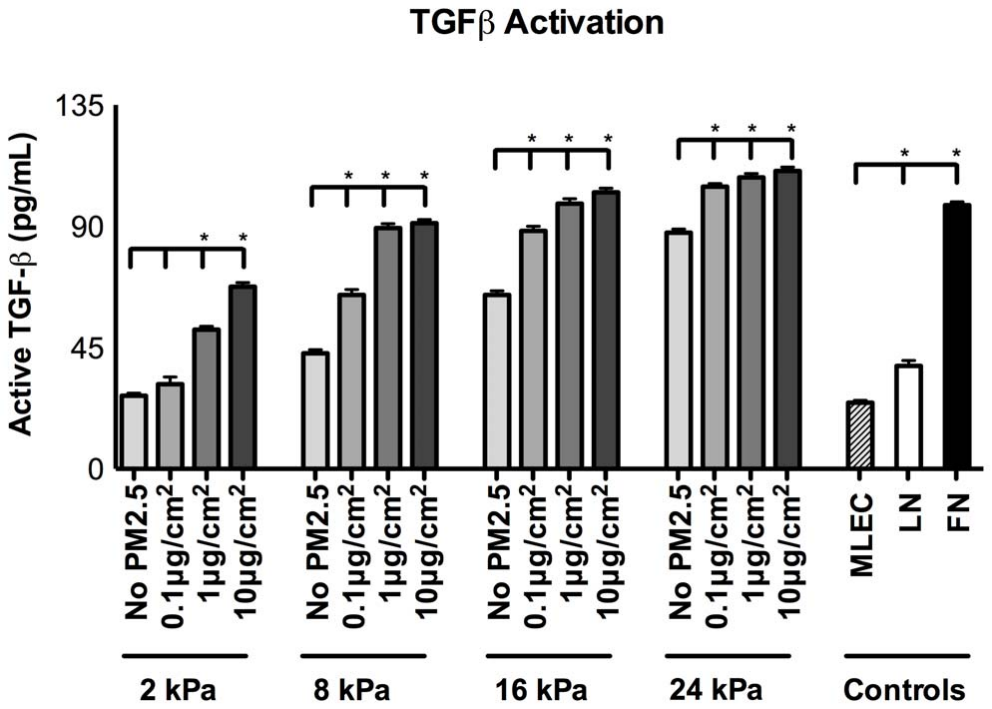
Stiffness-mediated activation of TGFβ is increased by addition of PM2.5. RLE-6TN cells were cultured for 5 days on substrates of increasing stiffness with or without the addition of each concentration of PM2.5 and levels of TGFβ activation were determined using the MLEC bioluminescence co-culture assay. Statistical significance is shown for each concentration of PM2.5 within its same substrate stiffness (* p<0.001).

Previous studies have shown that elevated levels of cell stiffness coincide with increased cell contractile forces, ultimately enabling the activation of latent TGFβ [27], [51]. Since we saw increases in cell-stiffness with the addition of PM2.5, we investigated if the addition of PM2.5 also resulted in differences in TGFβ activation, which could lead to further changes in ATII cell phenotype. To determine if addition of PM2.5 to alveolar cells on differing substrate stiffnesses induced TGFβ activation, the mink lung epithelial reporter cell (MLEC) bioluminescence co-culture assay was performed for each PM2.5 concentration. In order to confirm that substrate stiffness alone did not alter the MLEC sensitivity to active TGFβ, MLECs were cultured for 16 hours on each substrate stiffness with the addition of 200 ρg/ml active TGFβ and analyzed for changes in luminescence. Despite the changes in underlying substrate, the MLECs remained equally sensitive to the fixed amount of active TGFβ **(Figure S1)**. Similar to the cell morphology experiments, RLE-6TN cells were cultured on PA gels ranging from 2–24 kPa with or without the addition of each concentration of PM2.5. In controls without the addition of PM2.5, alveolar epithelial cells were found to increasingly activate TGFβ in response to increases in substrate stiffness. As expected, the control groups cultured on Fn-coated glass exhibited high levels of TGFβ activation, while those cultured on Ln-coated glass showed significantly less levels. When PM2.5 was added to the cultures for 5 days, the cells were found to activate significantly greater amounts of TGFβ in a dose dependent manner **(** **Figure 3** **)**.

Of particular interest was the comparison of TGFβ activation across the PM2.5 concentrations at 8 kPa, a physiologically relevant stiffness during the progression of human lung fibrosis. At this substrate stiffness with the addition of PM2.5, we observed an increase in TGFβ activation similar to what was observed on high substrate stiffness and Fn-coated glass without the PM2.5. PM2.5 addition to a solution of inactive TGFβ was found insufficient to activate TGFβ (**Figure 3** **, MLEC control**), strongly suggesting that increases in TGFβ activation observed are mediated by the cells. Statistical significance is for each experimental group compared within its same substrate stiffness.

### Exposure to PM2.5 results in increased TGFβ activation without cell contractility

The results thus far have indicated that exposure of RLE-6TN cells to PM2.5, most significantly at concentrations of 10 µg/cm^2^, result in significant changes to ATII cell phenotype. Briefly, ATII cells exhibit enhanced actin filament staining, cell stiffness, and TGFβ activation. These changes are likely dependent on cell contractility, allowing for a mechanically driven activation of TGFβ that subsequently drives the observed phenotypic changes [25]–[27], [29], [33], [34], [65]. Therefore, the MLEC assay that measures TGFβ activation was performed again with the same protocol but with the addition of the ROCK contractility inhibitor Y-27632 during the 5-day culture.

**Figure 4:**
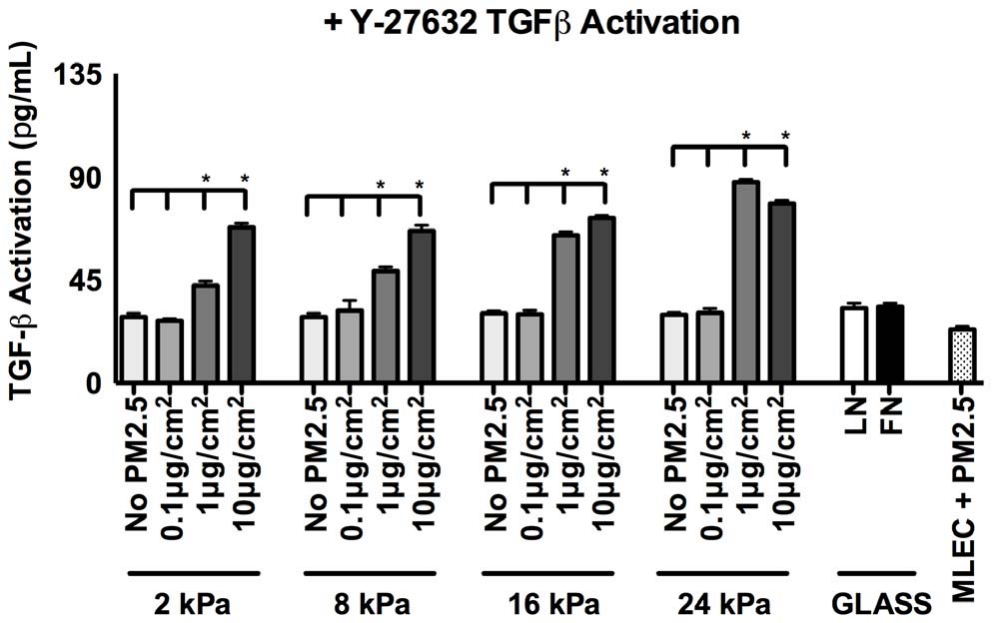
TGFβ activation is only partially mediated by cell contractility with PM2.5 exposure. RLE-6TN cells were cultured in each condition with the addition of the ROCK inhibitor, Y-27632, for 5 days and analyzed for TGFβ activation using the MLEC assay. Statistical significance is reported for differences between same substrate stiffness conditions compared to no PM2.5 control. (* p<0.001)

In the presence of the contractility inhibitor, Y-27632, cells that were not treated with PM2.5 activated low levels of TGFβ regardless of the substrate stiffness they were cultured on. However, there were significant increases in TGFβ activation with the addition of 10 µg/cm^2^ PM2.5 on all substrate stiffnesses **(** **Figure 4** **)**. These results suggest that inhibition of cell contractility is sufficient to inhibit substrate mediated TGFβ activation, but not sufficient to inhibit TGFβ activation mediated by the exposure to PM2.5. A control where PM2.5 was added to a solution of inactive TGFβ was found insufficient to activate TGFβ (**Figure 4** **, MLEC control**), again suggesting that these increases in TGFβ activation observed due to PM2.5 are still mediated by the cells, just not by cell contractility. This data suggests a second, non-mechanical mechanism of TGFβ activation that occurs when the cells are exposed to PM2.5.

### Exposure of RLE-6TN cells to PM2.5 results in increased levels of ROS

**Figure 5:**
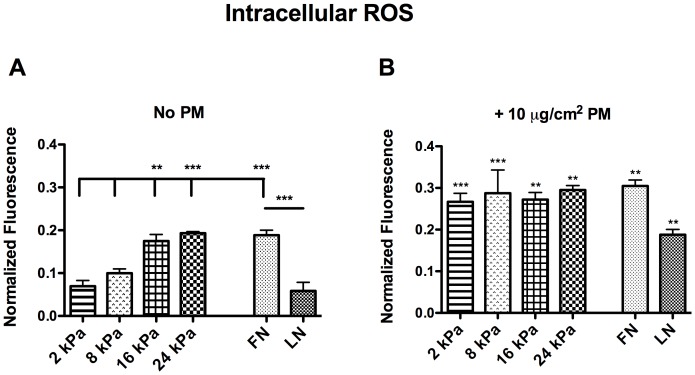
Exposure of RLE-6TN cells to increased stiffness and PM2.5 increases intracellular ROS. RLE-6TN cells were cultured on PA gels of increasing substrate stiffness with either no PM2.5 (A) or 10µg/cm^2^ PM2.5 (B) and intracellular ROS levels were measured by the DCFH_2_-DA oxidation assay. Three independent triplicate experiments were performed and statistical significance is shown between substrates (A) and in comparison to the matched no PM2.5 controls (B). ** (p<0.01) and *** (p<0.001).

It was hypothesized that the observed additional activation of TGFβ when cells were exposed to PM2.5 was due to intracellular production of ROS, ultimately leading to increased TGFβ activation [24], [74]. In order to explore this hypothesis, RLE-6TN cells were cultured on PA gels of increasing substrate-stiffness with or without exposure to 10 µg/cm^2^ of PM2.5 and analyzed by the DCFH_2_-DA assay, which measures total levels of intracellular ROS. When RLE-6TN cells were cultured on increasing substrate stiffnesses in the absence of PM2.5 there was a significant increase in ROS solely due to the underlying substrate, supporting previous studies [72] **(** **Figure 5A** **)**. Interestingly, the levels of ROS at 8 kPa, the stiffness most closely matching a remodeling lung, were not significantly higher than those seen on a soft substrate. However, when the cells were cultured with the addition of PM2.5, there was a significant increase in ROS observed on all substrates, including Fn- and Ln-coated glass **(** **Figure 5B** **)**. This suggests that increased tissue stiffness commonly seen in PF patients likely results in increased intracellular ROS levels and that exposure of the distal lung to PM2.5 significantly increases these levels of ROS. Furthermore, the data show similar levels of ROS across all substrate stiffnesses when treated with PM **(** **Figure 5B** **)**. Given that the measured fluorescence values were well within the range of detection for the assay, this data suggest that the cells may only be capable of producing a finite level of ROS, and that this maximum was approached.

Additionally, several studies to date have suggested antioxidants including N-acetyl-cysteine (NAC), may be effective in decreasing levels of ROS within lung tissue [75]–[77]. Therefore, it was explored if treatment with NAC was able to decrease the ROS production observed due to the exposure of cells to PM2.5. Similar to the previous experiment, RLE-6TN cells were cultured on increasing substrate stiffnesses with or without the addition of PM2.5. Additionally, groups with PM2.5 added were treated with 5 µM NAC. For each of the experimental groups, treatment with NAC abolished the levels of intracellular ROS observed with exposure to PM2.5 **(Figure S2)**. Notably, on higher substrate stiffnesses, i.e. 16 and 24 kPa, these antioxidants were able to reduce the ROS levels below the levels seen without the addition of PM2.5. This suggests that substrate stiffness alone is capable of inducing increases in intracellular ROS, and that this effect can be mitigated by treatment with an antioxidant.

### Stiffness-mediated ROS production is TGFβ dependent

**Figure 6:**
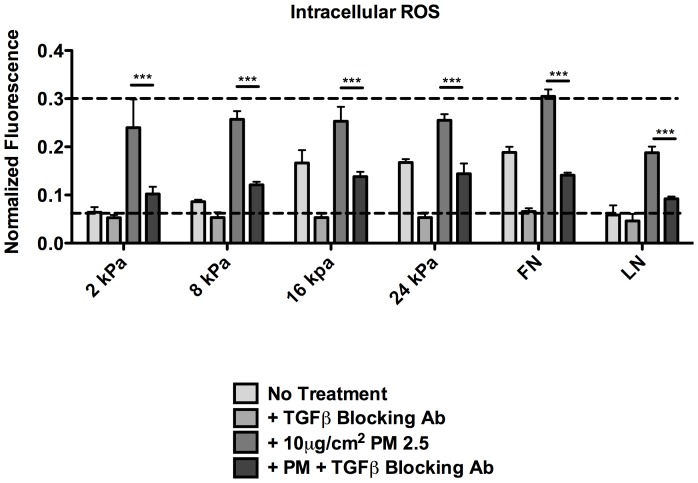
Stiffness mediated ROS production is TGFβ dependent. RLE-6TN cells were cultured on PA gels of increasing stiffness with or without the addition of 10 µg/cm^2^ and exposure to a TGFβ blocking antibody for 5 days. Levels of intracellular ROS were measured by the DCFH_2_-DA oxidation and assay. Three independent triplicate experiments were performed and statistical significance is shown between the PM2.5 groups with or without the TGFβ antibody. *** (p<0.001)

The previous data suggest that the levels of ROS increase as a function of matrix rigidity in the absence of treatment with PM2.5. Given previous literature that has shown an intimate link between ROS and TGFβ [24], [74], [78], we explored if this increase in ROS was TGFβ dependent. RLE-6TN cells were cultured with or without 10 µg/ml PM2.5 as well as with 10 µg/ml of a TGFβ neutralizing antibody for five days and analyzed for ROS production by the DCFH_2_-DA assay. When cells were cultured in the absence of PM and exposed to the TGFβ neutralizing antibody, the level of ROS production was reduced to baseline (i.e. Fn-2 kPa and Ln-glass) **(** **Figure 6** **)**, indicating that the increase in ROS due to substrate stiffness is a result of increased levels of active TGFβ. Additionally, when cells were treated with PM2.5 in conjunction with the neutralizing antibody there was a significant decrease in intracellular ROS, but not to the level seen without PM. Notably, this effect is most prominent at the lower substrate stiffness (2 kPa and 8 kPa), further suggesting that the cells may reach a maximal possible level of ROS production.

### Combinatorial treatments diminish increased levels of TGFβ activation

Previous data and the experiments thus far have strongly suggested that increased substrate stiffness is able to induce cell contractility leading to mechanical activation of TGFβ, resulting in downstream changes to ATII cell phenotype. Furthermore, when alveolar epithelial cells are exposed to PM2.5 there were further increases observed in TGFβ activation beyond that seen on increased substrate stiffness alone. Finally, exposure to PM2.5 resulted in increased intracellular ROS levels, which could be mitigated, but not abolished by a neutralizing TGFβ antibody. Therefore, we sought to determine if treatment with NAC could decrease the levels of TGFβ activation to a level seen on Ln-coated glass. Importantly, active TGFβ is a disulfide-linked homodimer, and NAC is capable of reducing disulfide bonds. Therefore, we first evaluated any differences in MLEC sensitivity to active TGFβ when also exposed to NAC. MLEC were cultured for 16 hours with a 200 ρg/ml TGFβ and 5 µM NAC combination and demonstrate a small, nonsignificant, but consistent decrease in the level of luciferase. When treated with 200 ρg/ml active TGFβ and NAC, the luciferase reading corresponded to an approximate value of 190 ρg/ml **(Figure S3)**. This was taken into consideration for the following experiments.

**Figure 7:**
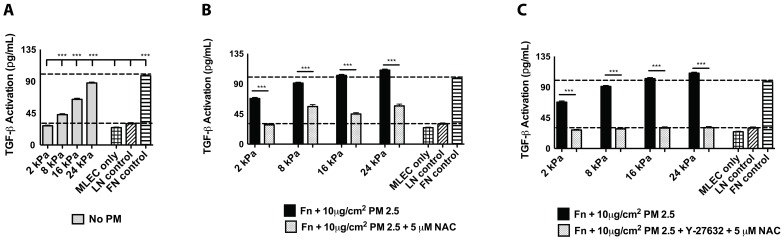
Combinatorial treatment with the ROCK inhibitor, Y-27632, and NAC restore low levels of TGFβ activation seen on epithelial maintaining substrates. RLE-6TN cells were cultured on increasing substrate stiffnesses (A) with 10 µg/cm^2^ PM2.5 (B) with or without treatment with the antioxidant NAC and levels of TGFβ activation measured . RLE-6TN cells were treated with a combination of the Y-27632 ROCK inhibitor and NAC and TGFβ activation measured (C). Three independent triplicate experiments were performed and statistical significance is shown between substrate stiffnesses (A) and to same substrate stiffness PM controls (B,C) *** (p<0.001).

RLE-6TN cells were cultured on increasing substrate stiffnesses with or without the addition of 10 µg/cm^2^ PM. Cells treated with PM were also exposed to 5 µM NAC. TGFβ activation levels were measured using the MLEC assay. RLE-6TN cells cultured in the absence of PM showed the predicted increase in TGFβ due to matrix rigidity, and a subsequent increase when exposed to PM **(** **Figure 7** **A,B)**. RLE-6TN cells that were cultured with PM2.5 and NAC showed a significant decrease in levels of TGFβ activation compared to cells cultured only with PM2.5. However, there was still incomplete reduction to the levels seen from cells cultured on Fn-2 kPa or Ln-coated glass **(** **Figure 7B** **)**. Therefore, we explored if treatment with the ROCK contractility inhibitor in conjunction with NAC could completely reduce levels of TGFβ activation. It was observed that a combinatorial treatment that increased antioxidants and blocked cell contractility was able to reduce TGFβ activation to levels similar to cells cultured on Ln despite the substrate stiffness of Fn-coated PA surfaces **(** **Figure 7C** **)**. These results taken together strongly suggest that increased tissue stiffness results in increased cell contractility which mechanically activates TGFβ; while exposure to PM2.5 further increases this TGFβ activation by increasing intracellular ROS. These events appear to have an additive effect on the activation of TGFβ, despite the underlying substrate stiffness and result in the significant changes observed in ATII cell phenotype.

## Discussion

TGFβ has been implicated as a major regulator during the progression of invasive pathologies such as PF. Therefore, it is imperative to understand how changes in the microenvironment during different pathological conditions affect TGFβ in order to give insight for future therapeutic options. Several studies have previously shown that the micromechanical environment that alveolar epithelial cells and resident fibroblasts interact with is a strong driving force for activating TGFβ . Here we show that in addition to increased tissue stiffness, exposure to environmental stimuli, such as PM2.5, is involved in further increasing TGFβ and driving changes to alveolar epithelial cell phenotype. Here we observe that PM2.5 is involved in the increase in epithelial cell cortical stiffness, an indirect measure of cell contraction, which enables mechanical activation the cytokine TGFβ. Our data show that increases in epithelial cell cortical stiffness coincide with increased levels of TGFβ activation, and an increase in elongated cells that exhibit enhanced actin staining. Experiments were performed where cell contractility was blocked by the ROCK inhibitor Y-27632 and TGFβ activation was measured by the MLEC assay. Interestingly, when cells were treated with 10 µg/cm^2^ PM2.5, inhibition of cell contractility was able to decrease TGFβ activation levels, but not to the level seen without PM2.5 exposure. This indicates that there is a second mechanism, independent of cell contraction, with which TGFβ is activated in response to PM2.5 exposure.

The alveolar epithelium forms a continuous, highly regulated physical barrier, which serves as a protector against inhaled environmental agents, including PM2.5. When epithelial cells become elongated, the tight cell junctions commonly seen in the epithelium are lost, likely resulting in decreased protection against inhaled agents. PM2.5 has been implicated in increasing levels of ROS and previous studies have shown that the release of ROS can activate TGFβ, [38], [43], [45], [79]. Additionally, TGFβ itself is known to be able to induce ROS production as part of its signal transduction pathway. Therefore, ROS were determined to be a likely culprit for the further increase in TGFβ activation that was observed that could not be abrogated by the inhibition of cell contractility. First, total levels of intracellular ROS with or without the addition of 10 µg/cm^2^ PM2.5 were measured on substrate stiffnesses ranging from 2 to 24 kPa. Significant increases in ROS were observed on all substrate stiffnesses when cells were exposed to PM2.5. Interestingly, as substrate stiffness was increased, there was a decreased difference between the non-treated and PM2.5 added groups, showing that substrate stiffness alone has an effect on increasing ROS levels. Furthermore, when cells were exposed to PM2.5, the levels of ROS production were similar despite the underlying matrices, suggesting that the cells may have a finite level of ROS they are capable of producing, and that this level was approached. We explored if the increase in ROS due to matrix rigidity alone was a function of increased amounts of active TGFβ by repeating the ROS assay in the presence of a TGFβ neutralizing antibody. It was shown that neutralizing TGFβ could eliminate the increased ROS due to substrate rigidity, but not due to exposure to PM2.5. Next, it was explored if treatment with the common antioxidant, NAC, could decrease the levels of ROS observed with PM2.5 exposure. When the cells were treated with NAC in conjunction with PM2.5 exposure, ROS levels were significantly decreased. Notably, on stiffer substrates, the antioxidant treatment decreased intracellular ROS to levels lower than seen on the matched substrate stiffness without the addition of PM2.5. This data further confirmed that substrate stiffness alone is capable of increasing levels of ROS.

A previous pulmonary fibrosis drug trial utilized treatment with NAC showed some benefit in delaying the progression of PF, but was not able to restore lung function or reverse any damage currently present [39]. From the data presented here, we conclude that NAC treatment alone was not a fully successful treatment since it has clearly been shown that the mechanical environment is a crucial mediator of TGFβ activation, alveolar cell phenotype changes, and the onset of a fibrotic scar. Therefore, in a final experiment, it was explored if combinatorial treatment with the antioxidant NAC in conjunction with the ROCK contractility inhibitor, could mediate the significantly increased levels of TGFβ activation previously observed with epithelial cells cultured on increased substrate stiffness and exposed to PM2.5. When alveolar epithelial cells were cultured with PM2.5 and treated with NAC as well as Y-27632, levels of TGFβ were decreased to levels seen from cells cultured on Ln, the known epithelial maintaining substrate. This level of TGFβ activation was seen despite the stiffness of the substrate that cells were cultured on. This final piece of data strongly suggests that TGFβ activation is mediated by a combination of mechanical stimuli, i.e. tissue stiffness, and changes in the intracellular levels of ROS.

This work shows that alveolar epithelial cells exhibit increased cell stiffness, TGFβ activation, and changes in their phenotype in response to PM2.5 exposure. These results highlight the importance of studying not only the micromechanical environment in lung disease but also the exposure to environmental injury adjuvants in diseased lung models. Previous findings have indicated that increased tissue stiffness is able to lead to increased cell contraction and TGFβ activation and represents one of the initial stages of pulmonary fibrosis [16], [18], [25], [26], [34], [70]. Here these findings are expanded and show that in addition to the delicate balance between ECM stiffness and TGFβ, that exposure to environmental stimuli such as PM2.5 enhances TGFβ activation through increasing levels of intracellular ROS. Specifically, it appears that there is an additive effect on TGFβ activation from mechanical stimuli and intracellular ROS production in response to environmental stimuli, resulting in significantly increased changes to alveolar epithelial cell phenotype.

Finally, it is shown that by controlling *both* the micromechanical and biochemical environment through blocking cell contractility and reducing intracellular levels of ROS, that TGFβ activation can be reduced to levels typically seen in ‘healthy’ epithelial cells. This work suggests that PM2.5 is able to significantly increase the levels of TGFβ activation and may have a role in driving a pre-existing fibrotic phenotype in pulmonary cells. Therefore, approaches to treat pulmonary fibrosis must focus on controlling both the micromechanical stimuli presented to alveolar epithelial cells while also controlling the oxidative effect of increased ROS. Future work should focus on further elucidation of the pathways that both tissue stiffness and PM2.5 engage to initiate changes to alveolar cell phenotype and additionally how these pathways intersect in order to develop novel treatment therapies for pulmonary fibrosis.

## Supporting Information

Figure S1 **MLEC are equally responsive to active TGFβ on increasing substrate stiffnesses.** MLEC were cultured for 16 hours on substrates of increasing stiffness for 16 hours with the addition of 200 ρg/ml active TGFβ and analyzed for any changes in luciferase response (A). Three independent triplicate experiments were performed.
(TIF)

Figure S2 **Addition of the antioxidant NAC restores low levels of ROS.** RLE-6TN cells were cultured on increasing substrate stiffnesses for 5 days with or without 10µg/cm^2^ PM2.5 and 5µM NAC and levels of intracellular ROS measured by the DCFH_2_-DA oxidation assay. Three independent triplicate experiments were performed and statistical significance shown between the PM2.5 treated groups ***.
(TIF)

Figure S3 **MLEC show small decrease in response to active TGFβ when treated with NAC.** MLEC were cultured for 16 hours on increasing substrate stiffnesses with the addition of 200 ρg/mL active TGFβ and 5µM NAC and analyzed for changes in luciferase response. Three independent triplicate experiments were performed.
(TIF)

Table S1 **South Dekalb Elemental Analysis.** Filters collected on given dates were analyzed for specific elemental analysis and are reported in µg/m^3^.
(TIFF)
