# Supplementary figures and images for: Environmental Particulate (PM2.5) Augments Stiffness-Induced Alveolar Epithelial Cell Mechanoactivation of Transforming Growth Factor Beta
Source: PLoS One. 2014 Sep 16;9(9):e106821. doi: 10.1371/journal.pone.0106821 (PMC4167324; doi:10.1371/journal.pone.0106821)

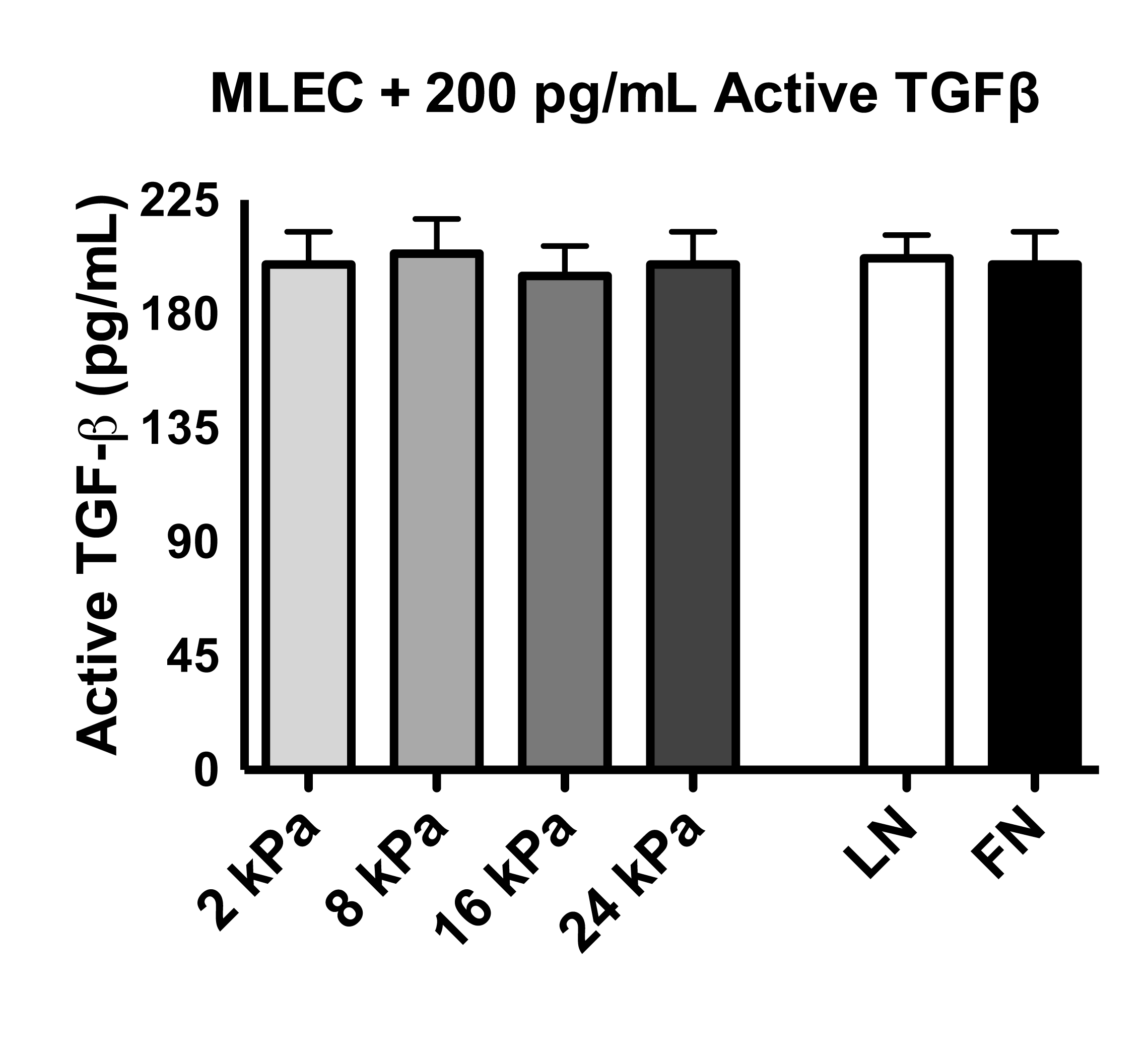

Supplement: Figure S1 — MLEC are equally responsive to active TGFβ on increasing substrate stiffnesses. MLEC were cultured for 16 hours on substrates of increasing stiffness for 16 hours with the addition of 200 ρg/ml active TGFβ and analyzed for any changes in luciferase response (A). Three independent triplicate experiments were performed. (TIF) [file pone.0106821.s001.tif]

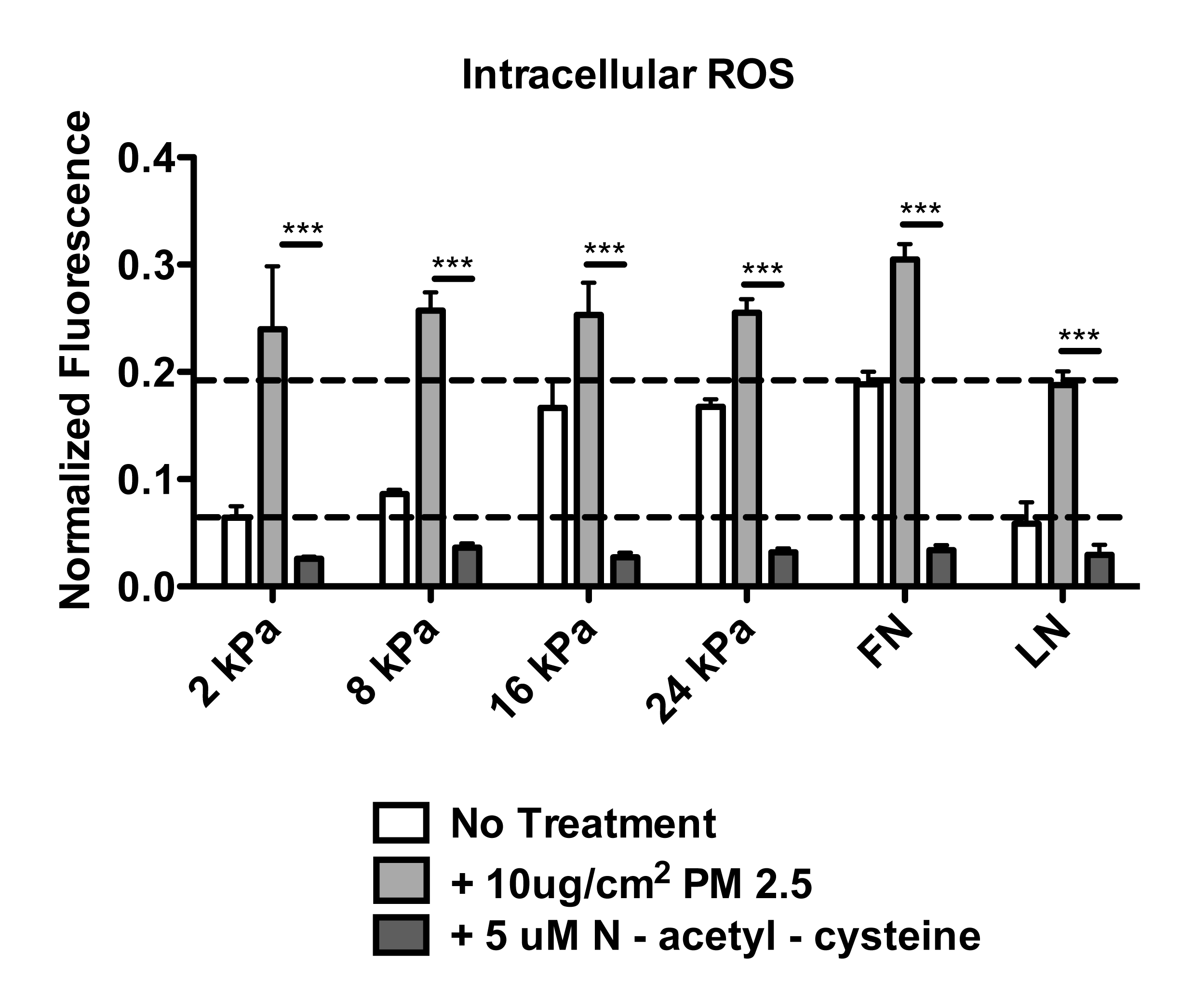

Supplement: Figure S2 — Addition of the antioxidant NAC restores low levels of ROS. RLE-6TN cells were cultured on increasing substrate stiffnesses for 5 days with or without 10µg/cm2 PM2.5 and 5µM NAC and levels of intracellular ROS measured by the DCFH2-DA oxidation assay. Three independent triplicate experiments were performed and statistical significance shown between the PM2.5 treated groups ***. (TIF) [file pone.0106821.s002.tif]

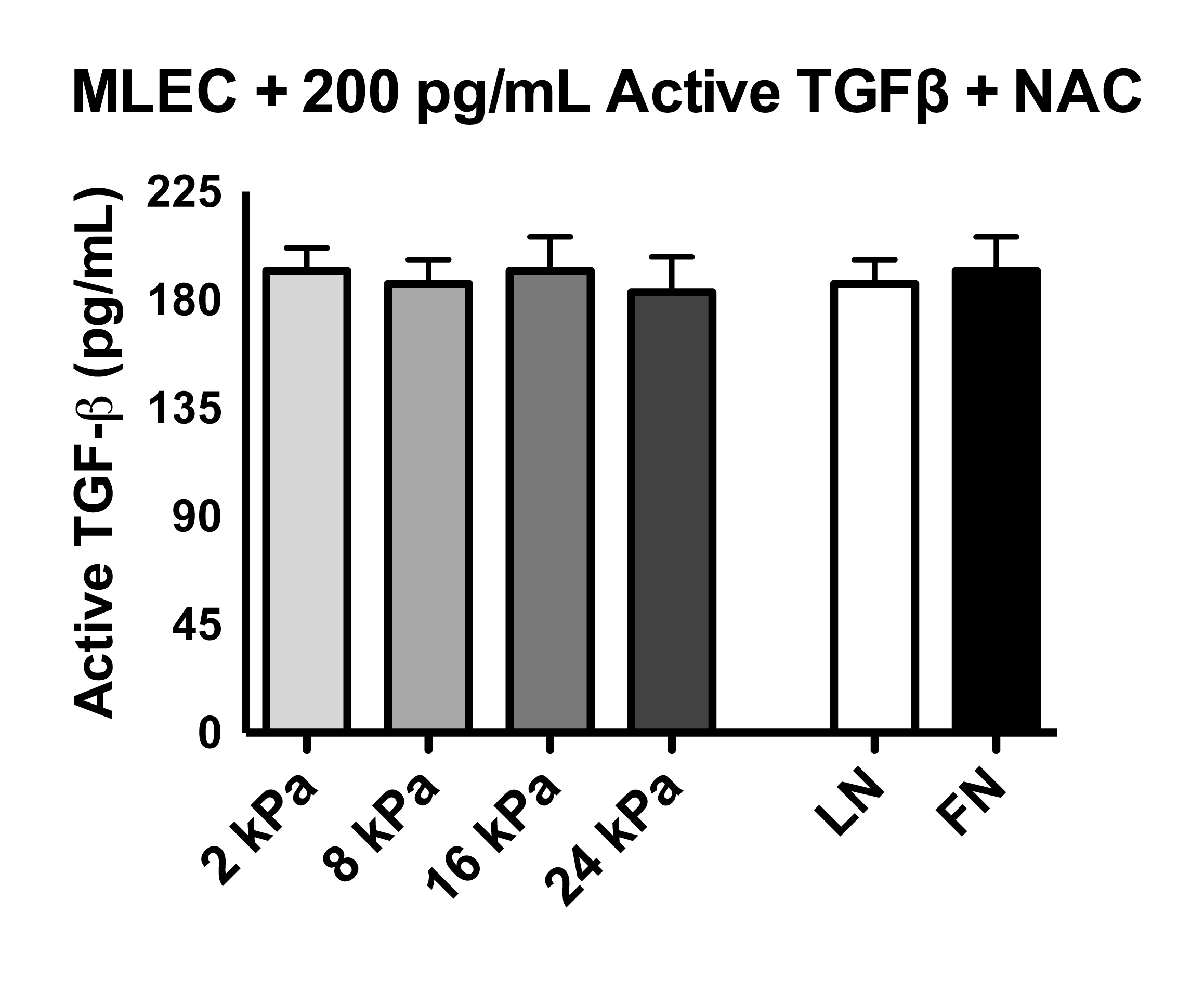

Supplement: Figure S3 — MLEC show small decrease in response to active TGFβ when treated with NAC. MLEC were cultured for 16 hours on increasing substrate stiffnesses with the addition of 200 ρg/mL active TGFβ and 5µM NAC and analyzed for changes in luciferase response. Three independent triplicate experiments were performed. (TIF) [file pone.0106821.s003.tif]

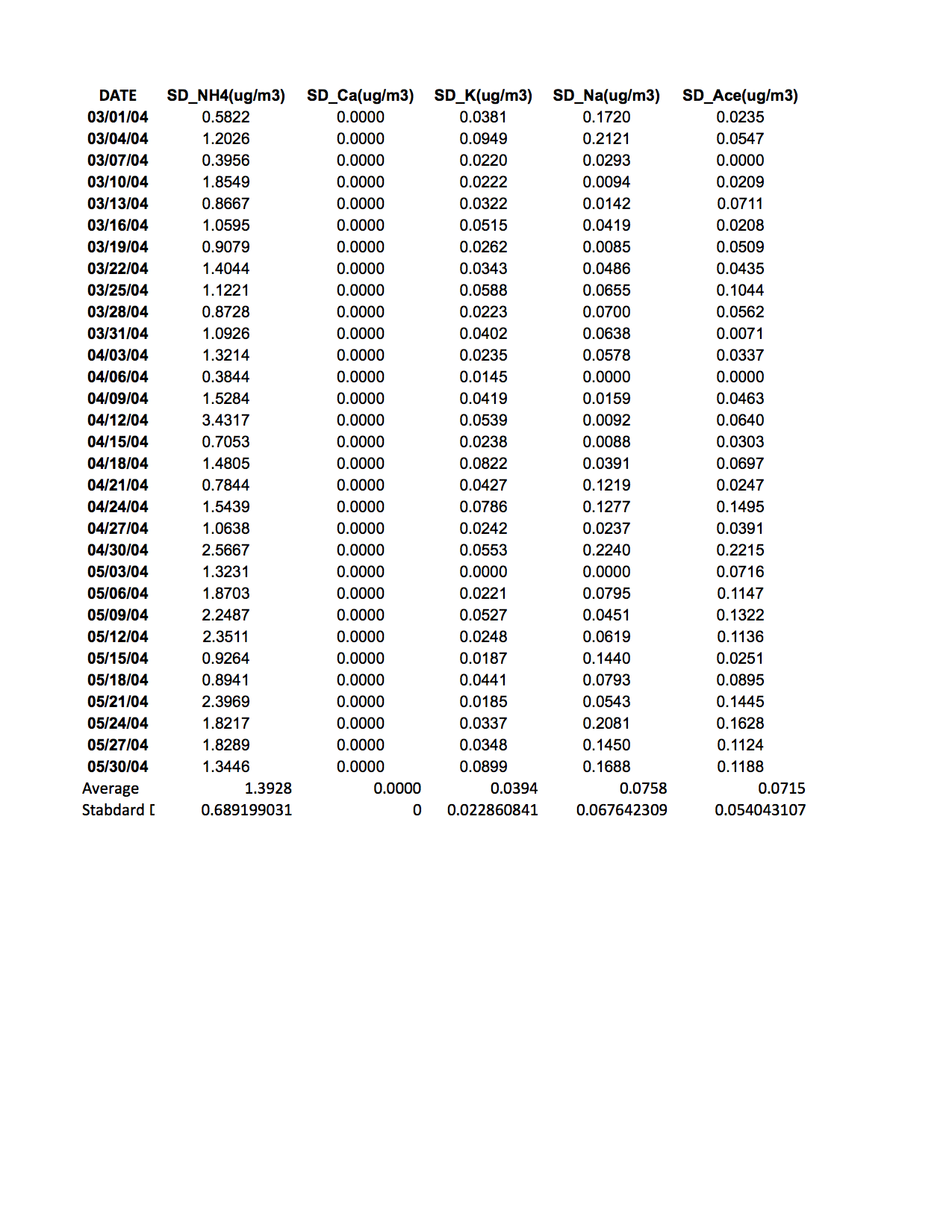

Supplement: Table S1 — South Dekalb Elemental Analysis. Filters collected on given dates were analyzed for specific elemental analysis and are reported in µg/m3. (TIFF) [file pone.0106821.s004.tiff]
